# Engineering a mevalonate pathway in *Halomonas bluephagenesis* for the production of lycopene

**DOI:** 10.3389/fmicb.2022.1100745

**Published:** 2023-01-16

**Authors:** Qixuan Su, Ping Cheng, Jiyuan Sun, Yulin Zhang, Yang Zheng, Xiao-Ran Jiang, Xiancai Rao

**Affiliations:** ^1^Cancer Center, Medical Research Institute, Southwest University, Chongqing, China; ^2^Department of Microbiology, College of Basic Medical Sciences, Army Medical University (Third Military Medical University), Chongqing, China

**Keywords:** *Halomonas bluephagenesis*, carotenoid products, lycopene, mevalonate pathway, lycopene biosynthesis

## Abstract

**Introduction:**

Red-colored lycopene has received remarkable attention in medicine because of its antioxidant properties for reducing the risks of many human cancers. However, the extraction of lycopene from natural hosts is limited. Moreover, the chemically synthesized lycopene raises safety concerns due to residual chemical reagents. *Halomonas bluephagenesis* is a versatile chassis for the production of fine chemicals because of its open growth property without sterilization.

**Methods:**

A heterologous mevalonate (MVA) pathway was introduced into *H. bluephagenesis* strain TD1.0 to engineer a bacterial host for lycopene production. A pTer7 plasmid mediating the expression of six MVA pathway genes under the control of a phage *P_Mmp1_* and an *Escherichia coli P_trc_* promoters and a pTer3 plasmid providing lycopene biosynthesis downstream genes derived from *Streptomyces avermitilis* were constructed and transformed into TD1.0. The production of lycopene in the engineered *H. bluephagenesis* was evaluated. Optimization of engineered bacteria was performed to increase lycopene yield.

**Results:**

The engineered TD1.0/pTer7-pTer3 produced lycopene at a maximum yield of 0.20 mg/g dried cell weight (DCW). Replacing downstream genes with those from *S. lividans* elevated the lycopene production to 0.70 mg/g DCW in the TD1.0/pTer7-pTer5 strain. Optimizing the *P_Mmp1_* promoter in plasmid pTer7 with a relatively weak *P_trc_* even increased the lycopene production to 1.22 mg/g DCW. However, the change in the *P_trc_* promoter in pTer7 with *P_Mmp1_* did not improve the yield of lycopene.

**Conclusion:**

We first engineered an *H. bluephagenesis* for the lycopene production. The co-optimization of downstream genes and promoters governing MVA pathway gene expressions can synergistically enhance the microbial overproduction of lycopene.

## Introduction

Lycopene (C_40_H_56_, 536.85 Da) is a common carotenoid responsible for the red color in some plants, algae, fungi, and bacteria (Andrewes et al., 1976). It acts as a principal chemical in carotenoid production (Sharma et al., 2016). Other carotenoid pigments, such as β-carotene, astaxanthin, and canthaxanthin, are synthesized from lycopene (Rad et al., 2012). The straight-chain hydrocarbon of lycopene contains 11 double conjugated bonds and two nonconjugated double bonds, enabling its antioxidant properties (Shi and Le Maguer, 2000; Ma et al., 2016). Lycopene has received remarkable attention in medicine because of its excellent effects on human disease control, such as tumor suppression (Aghajanpour et al., 2017; Grether-Beck et al., 2017) and cardiovascular system protection (Müller et al., 2016). Clinical studies have shown that dietary lycopene can inhibit the spread of established tumors, including prostate, colorectal, gastric, pancreatic, and lung cancers (Singh and Goyal, 2008). Thus, the large-scale production of lycopene is vital to meeting the growing demand for natural additives or drugs.

Lycopene has largely been extracted from plants with nonpolar solvents, such as tomatoes and carrots (Rad et al., 2012). However, the unstable supply of raw plant sources and the finite content make lycopene production costly (Saini and Keum, 2018). The chemical synthesis of lycopene is feasible. However, it often raises safety concerns because of chemical reagent residue (Ernst, 2002; Reynaud et al., 2011). Recently, researchers have devoted much effort to lycopene production *via* fermentation with microbial chassis cells, including *Escherichia coli* and *Saccharomyces cerevisiae* (Rad et al., 2012; Li et al., 2019). Two biosynthetic pathways, the mevalonate (MVA) pathway and the 2C-methyl-D-erythritol 4-phosphate (MEP) pathway, are characterized as the upstream module involved in lycopene synthesis in organisms (Rohmer et al., 1993; Yang et al., 2012). Eukaryotes commonly use the MVA pathway to synthesize lycopene, whereas most bacteria carry the MEP pathway (Bloch et al., 1959; Moise et al., 2014; Li et al., 2018; Ma Y. R. et al., 2019). Compared with the MEP pathway, the MVA pathway is often economical and highly efficient (Cunningham et al., 1994). The MVA synthetic pathway starts from acetyl-CoA to produce isoprene pyrophosphate (IPP) under the catalysis of specific enzymes, including acetyl-CoA acetyltransferase (MvaE), hydroxymethylglutaryl-CoA synthase (MvaS), mevalonate kinase (Mvk), mevalonate 5-phosphate kinase (Pmk), and mevalonate pyrophosphate decarboxylase (MvaD; Jervis et al., 2019). The MEP pathway begins from pyruvate and D-glyceraldehyde 3-phosphate to synthesize IPP (Moise et al., 2014; Li et al., 2018). IPP can be isomerized to dimethylallyl diphosphate (DMAPP) *via* isomerase (IDI). IPP and DMAPP are substrates for final lycopene production *via* the downstream enzymes, including geranylgeranyl diphosphate synthase (CrtE), phytoene synthase (CrtB), and lycopene synthase (CrtI; Sun et al., 2014).

As for metabolic engineering, heterogeneous genes involved in both upstream and downstream, modules of lycopene biosynthesis can be selected and constructed into a single chassis to generate a microbial host of interest (Jervis et al., 2019). Many optimizing strategies, including precursor substance increase, synthetic gene expression enhancement, competitive pathway gene inhibition, and by-product synthesis reduction, have been performed to increase lycopene yield (Kim and Keasling, 2001; Wang et al., 2012; Yan et al., 2013; Wang et al., 2014). In the present study, a heterologous MVA pathway carrying *mvaE* and *mvaS* genes derived from *Enterococcus faecalis* (*Ef*), *mvaK1*, *mvaK2*, and *mvaD* genes originated from *Streptococcus pneumoniae* (*Sp*), and *idi* gene derived from *E. coli* (*Ec*) was constructed in the pTer7 plasmid that was subsequently transformed into *Halomonas bluephagenesis* strain TD1.0, a non-carotenogenic bacterium that can grow pollution-free in unsterilized seawater (Manachini and Fortina, 1998; Yin et al., 2014; Zhao et al., 2017; Jervis et al., 2019). After the co-transformation of the pTer3 plasmid containing *crtE*, *crtB*, and *crtI* lycopene biosynthesis downstream genes from *Streptomyces avermitilis* (*Sa*), the engineered TD1.0/pTer7-pTer3 could successfully produce lycopene by a yield of 0.20 mg/g dried cell weight (DCW). Replacing downstream genes with those from *S. lividans* (*Sl*) considerably increased the lycopene production to 0.70 mg/g DCW. Further optimization of promoters involved in the MVA pathway even elevated lycopene yield to 1.22 mg/g DCW, which was a 6.1-fold increase relative to that produced by TD1.0/pTer7-pTer3. Our data showed that an engineered *H. bluephagenesis* is successfully generated for lycopene production. Changing the source of metabolism-responsible genes and optimization module gene promoters can effectively promote the microbial production of lycopene.

## Materials and methods

### Bacterial strains and growth conditions

The bacterial strains used in this study are listed in Supplementary Table 1. *H. bluephagenesis* TD1.0 (CGMCC 4353), a novel T7-like RNA polymerase-integrated derivative of the wild-type *H. bluephagenesis* strain TD01 that was isolated from Aydingol Lake of Xinjiang province in China (Zhao et al., 2017), was used as a chassis. *Escherichia coli* S17-1 was used to manipulate plasmids and served as the conjugation donor (Simon, 1984). *E. coli* strain was cultured at 37°C in the Luria-Bertani (LB) medium (Analytical reagent, Oxoid, England) or plated on LB agar for 16 h. *H. bluephagenesis* TD1.0 and its derivatives were cultivated with 60-LB medium (LB medium supplemented with 60 g/L NaCl) or 60-LB agar at 37°C for 16 h. Whenever necessary, the bacterial media were supplemented with chloramphenicol (Cm, 25 μg/mL), kanamycin (Kan, 50 μg/mL), or spectinomycin (Spe, 100 μg/mL) for maintaining plasmids.

### Plasmid construction

The plasmids used in this study are listed in Supplementary Table 1. All plasmids were constructed using Gibson Assembly (Gibson et al., 2009) with appropriate fragments obtained by polymerase chain reaction (PCR) with the primers mentioned in Supplementary Table 2. Taking the construction of pTer1 plasmid as an example, primers with 20–40 bp homologous regions were designed. A small DNA fragment (3,561 bp) carrying OriT/OriV was amplified from the pSEVA321 plasmid template with primer pairs ter1-3/R24. A large fragment (8,827 bp) containing the heterogeneous MVA pathway genes, including *EfmvaE*, *EfmvaS*, *SpmvaK1*, *SpmvaK2*, *SpmvaD*, and *Ecidi* under the control of two *E. coli P_trc_* promoters cloned in pMVA2 (Jervis et al., 2019), was also obtained by PCR using primers ter1-1/ter1-2. The PCR fragments were purified by a DNA purification kit (Sangon Biotech, China) and ligated with a Gibson Assembly kit [New England Biolabs (Beijing), China]. Then, the ligation product was electroporated into *E. coli* S17-1 competent cells and plated on LB agar supplemented with 25 μg/mL of Cm. After 16 h of incubation at 37°C, the Cm-resistant colonies were picked and cultured in LB broth with Cm at 37°C overnight. The plasmid was extracted with a plasmid extraction kit (Tiangen Biotech, China) and subjected to PCR verification and DNA sequencing. The relevant plasmid was designated as pTer1 (Supplementary Table 1). Other plasmids were constructed in the same way, using specific DNA templates and primers.

### Bacterial conjugation

*H. bluephagenesis* was unable to undergo chemical and electrical transformation; therefore, a conjugation was performed (Jiang et al., 2021). Briefly, the 20-LB medium (LB supplemented with 20 g/L NaCl) was used in the conjugation experiment to allow the surviving of *E. coli* donor cells. A single colony of *E. coli* S17-1 carrying certain plasmid was cultured in LB medium at 37°C for 16 h. After culture, *E. coli* cells were transferred to fresh LB (1% inoculation) and incubated at 37°C to achieve an optical density (OD) at 600 nm (OD_600_) of 0.5–0.6. The bacterial cells in 1 mL culture were recovered by centrifugation at 2,500 ×g for 5 min and resuspended with 50 μL of 20-LB without antibiotics. *H. bluephagenesis* TD1.0 was cultivated with 60-LB and prepared as mentioned above. *H. bluephagenesis* TD1.0 sample was 1:1 (vol/vol) mixed with the plasmid-carried *E. coli* S17-1 donor cells and inoculated into 20-LB agar without antibiotics. After 6 h of incubation at 37°C, the bacteria were collected and streaked onto 60-LB agar plate with appropriate antibiotics corresponding to the plasmid and cultured at 37°C for 16 h. The single colony was selected and cultured in 60-LB medium. The plasmid in the conjugated *H. bluephagenesis* TD1.0 was extracted and verified with PCR amplification.

### Bacterial growth curve

*H. bluephagenesis* TD1.0 or its derivatives were cultured in a small conical flask (100 mL) with 60-LB broth at 37°C overnight with shaking. On the next day, the culture was inoculated (1%) to 20 mL fresh 60-LB medium. A total of 50 μL culture was taken every hour and dropped in triplicate into the wells of a 96-well plate, and the OD_600_ value was detected by a FLUOstar Omega microplate reader (BMG Labtech). The growth curve was drawn by plotting OD_600_ values vs. culture time.

### Shake flask fermentation

Three colonies of *H. bluephagenesis* TD1.0 or its variants were taken from a 60-LB agar plate for each experiment, inoculated individually into 2 mL of 60-LB medium, and then cultured at 37°C for 12–16 h. Subsequently, the culture was transferred to 20 mL of 60-LB medium in a 100-mL conical flask (1% inoculum, vol/vol) and cultured at 37°C to an OD_600_ value of 0.6–0.8. Then, the secondary culture was inoculated into 50 mL fresh 60-MMG medium [Minimal medium containing 60 g/L NaCl, 0.05% urea (m/vol), 0.02% MgSO_4_ (m/vol), 1.0% Na_2_HPO_4_·12H_2_O (m/vol), 0.15% KH_2_PO_4_ (m/vol), 1.0% trace element solution I (vol/vol), 0.1% trace element solution II (vol/vol), and 0.1% yeast extract (m/vol)]. The pH of the solution was adjusted to 8.0–8.5 using 5 M NaOH. Trace element solution I comprises 0.5% Fe (III)-NH_4_-citrate (m/vol) and 0.2% CaCl_2_ (m/vol) dissolved in 1 M HCl; Trace element solution II contains 0.01% ZnSO_4_·7H_2_O (m/vol), 0.003% MnCl_2_·4H_2_O (m/vol), 0.03% H_3_BO_3_ (m/vol), 0.02% CoCl_2_·6H_2_O (m/vol), 0.003% NaMoO_4_·2H_2_O (m/vol), 0.002% NiCl_2_·6H_2_O (m/vol), and 0.001% CuSO_4_·5H_2_O (m/vol) dissolved in 1 M HCl. Glucose was added at a final concentration of 3% (m/vol) at the beginning of the experiment in a 500-mL conical flask (5% inoculum, vol/vol) and cultured at 37°C for 48 h with shaking. Then, 30 mL of fermentation culture was harvested and pelleted by centrifugation at 10,000 ×g for 10 min at 4°C in a sterile centrifuge tube (50 mL) that had been prepared and weighed. The supernatant was removed, the tube was sealed with plastic wrap and placed in a −80°C refrigerator for 1 h, then bacterial cells were treated with a lyophilizer at least 8 h. The total weight was weighed, and the dried cell weight (DCW) was calculated.

### High performance liquid chromatography analysis

After flask fermentation, 1 mL of the culture was centrifuged at 5,000 ×g for 5 min, and bacterial cells were resuspended and lysed with acetone solution (using 1% butylated hydroxytoluene), vortexed for 5 min. Then, the cell lysate was centrifuged at 10,000 ×g for 5 min, and the supernatant was filtered through a 0.22 μm filter (Millipore) into the liquid sample bottle. Lycopene standard was prepared and diluted with acetone for five concentration gradients (1.25, 2.50, 5.00, 10.00, and 20.00 mg/mL) to draw standard curves, as previously described (Ma T. et al., 2019). An high performance liquid chromatography (HPLC) system (Agilent 1,260 Infinity II; Agilent Technologies, United States) equipped with a C_18_ column (4.6 mm × 150 mm; 5 μm; Agilent Technologies, United States) was used. The following parameters were set for HPLC analysis: The flow rate was 1 mL/min, the column temperature was kept at 30°C, the detection wavelength was 470 nm, and the mobile phase ratios of the solvents methanol/acetonitrile/isopropanol were 42/42/16, respectively. The peak area of the sample was substituted into the standard curve, and the lycopene yield was computed.

### RT-qPCR determination

*H. bluephagenesis* TD1.0 or its derivatives were cultured with 60-LB broth at 37°C for 16 h. The overnight culture was inoculated into 2 mL of fresh 60-LB medium (1% inoculum, vol/vol) and cultured at 37°C. Diverse concentrations of IPTG were added when the OD_600_ reached 0.5–0.6. The bacterial cells were harvested and the total RNA was extracted using a RNAprep Pure Cell/Bacteria Kit (Tiangen, China). For each sample, three duplicate samples were prepared. The first cDNA was synthesized with a RevertAid First Strand cDNA Synthesis Kit (Thermo Fisher Scientific). A quantitative polymerase chain reaction (qPCR) was conducted, and the relative expression level of each gene of interest was calculated as a ratio of its *C*_t_ value relative to that of the reference 16S rRNA gene.

### Statistical analysis

All results were analyzed with GraphPad Prism 8. Results derived from samples between two groups were treated with unpaired two-tailed Student’s *t*-test, and the difference between groups was calculated with analysis of variance (ANOVA). Each experiment was conducted at least three times, and the data were presented as mean ± standard deviations (SD). The statistically significant was considered as a *p*-value < 0.05.

## Results

### Construction of lycopene synthetic pathway in *Halomonas bluephagenesis*

**Figure 1:**
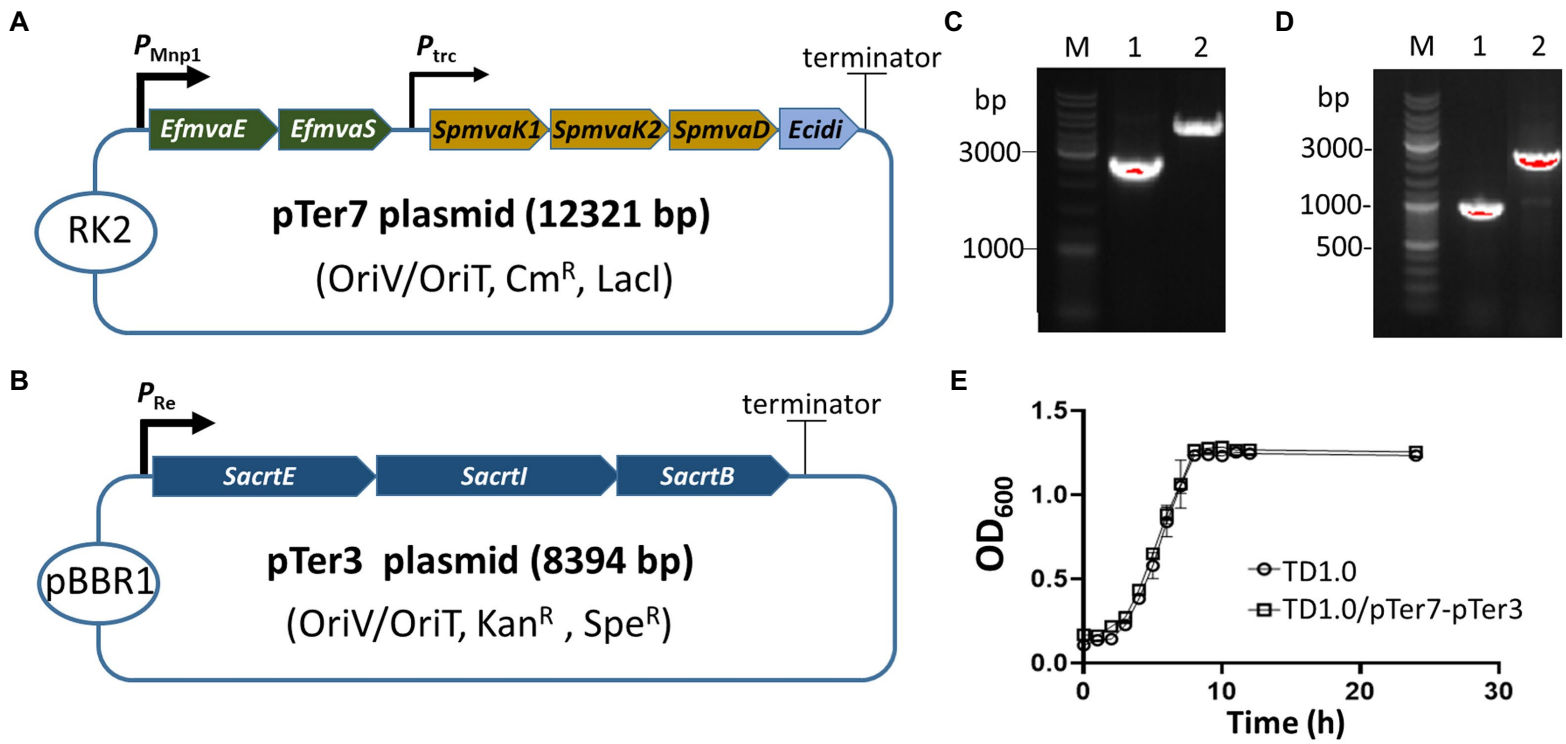
Construction and identification of the lycopene-producing *Halomonas bluephagenesis* strain TD1.0/pTer7-pTer3. **(A)** Schematic diagram of the construction of the pTer7 plasmid carrying genes involved in the MVA pathway. **(B)** Diagram representing the construction of pTer3 harboring *SacrtE*, *SacrtI*, and *SacrtB* genes. **(C)** PCR characterization of pTer7 and pTer3 plasmids in diverse *Escherichia coli* S17-1 strains. Lane 1, PCR products using jxr53-6/pMVA112 primers from the pTer7 template (approximately 2.6 kb). Lane 2, PCR products using F24/R24 primers from the pTer3 template (about 4.4 kb). The represented molecular size of the DNA Marker was indicated on the left. **(D)** PCR analysis of pTer7 and pTer3 plasmids in *H. bluephagenesis* strain TD1.0/pTer7-pTer3. Lane 1, PCR products using 24R/jxr53-6 primers from the pTer7 template (1.0 kb). Lane 2, PCR products using jxr53-5/jxr53-6 primers from the pTer3 template (2.0 kb). All primers used were listed in Supplementary Table 2. **(E)** The growth curves of *H. bluephagenesis* strains TD1.0 wild-type and TD1.0/pTer7-pTer3.

Two biosynthesis pathways (MVA and MEP) involved in lycopene production have been characterized in microorganisms, such as *E. coli* and *Pseudomonas putida* (Rohmer et al., 1993; Hernandez-Arranz et al., 2019). *H. bluephagenesis* has a potential MEP pathway for producing DMAPP from phosphoglyceraldehyde (Supplementary Figure 1). However, it lacks the downstream enzymes in the conversion of DMAPP to lycopene. The MEP pathway in *H. bluephagenesis* carries seven genes, and the long metabolic pathway may be tied to unknown physiological control elements to reduce the production efficiency of DMAPP. Therefore, a pTer1 plasmid was constructed by ligating a small fragment derived from the pSEVA321 vector and a large fragment amplified from the pMVA2 plasmid that carries microbial MVA pathway genes, including *EfmvaE*, *EfmvaS*, *SpmvaK1*, *SpmvaK2*, *SpmvaD*, and *Ecidi* (Jervis et al., 2019; Supplementary Figure 2). The *E. coli P_trc_* promoter upstream of *EfmvaE* and *EfmvaS* genes was replaced by a strong one (*P_Mmp1_*) derived from phage MmP1 (Zhao et al., 2017; Figure 1A). The resultant plasmid was designated as pTer7 (Supplementary Figure 3A). After the transformation into *E. coli* S17-1, the construction of pTer7 was verified *via* PCR amplification and DNA sequencing (Figure 1C; Supplementary Figure 3). Bacterial conjugation was performed using *E. coli* S17-1/pTer7 as a donor and *H. bluephagenesis* strain TD1.0 as the recipient bacterium. The successfully conjugated pTer7 in TD1.0 was further characterized by PCR amplification (Figure 1D).

We generated a pTer3 plasmid that carries the *crtE*, *crtI*, and *crtB* derived from *S. avermitilis* in the pSEV434 plasmid to introduce the downstream of the lycopene synthetic pathway to *H. bluephagenesis* strain TD1.0 (Supplementary Figure 1; Supplementary Table 1). The resultant pTer3 in *E. coli* S17-1 and *H. bluephagenesis* strain TD1.0 was extracted and verified *via* PCR amplification and DNA sequencing, respectively (Figures 1C,D; Supplementary Figure 4). *H. bluephagenesis* strain TD1.0/pTer7-pTer3 showed a similar growth rate to the wild-type TD1.0, indicating successful carriage of dual plasmids in *H. bluephagenesis* strain TD1.0 (Figure 1E).

### Production of lycopene in *Halomonas bluephagenesis* TD1.0/pTer7-pTer3

**Figure 2:**
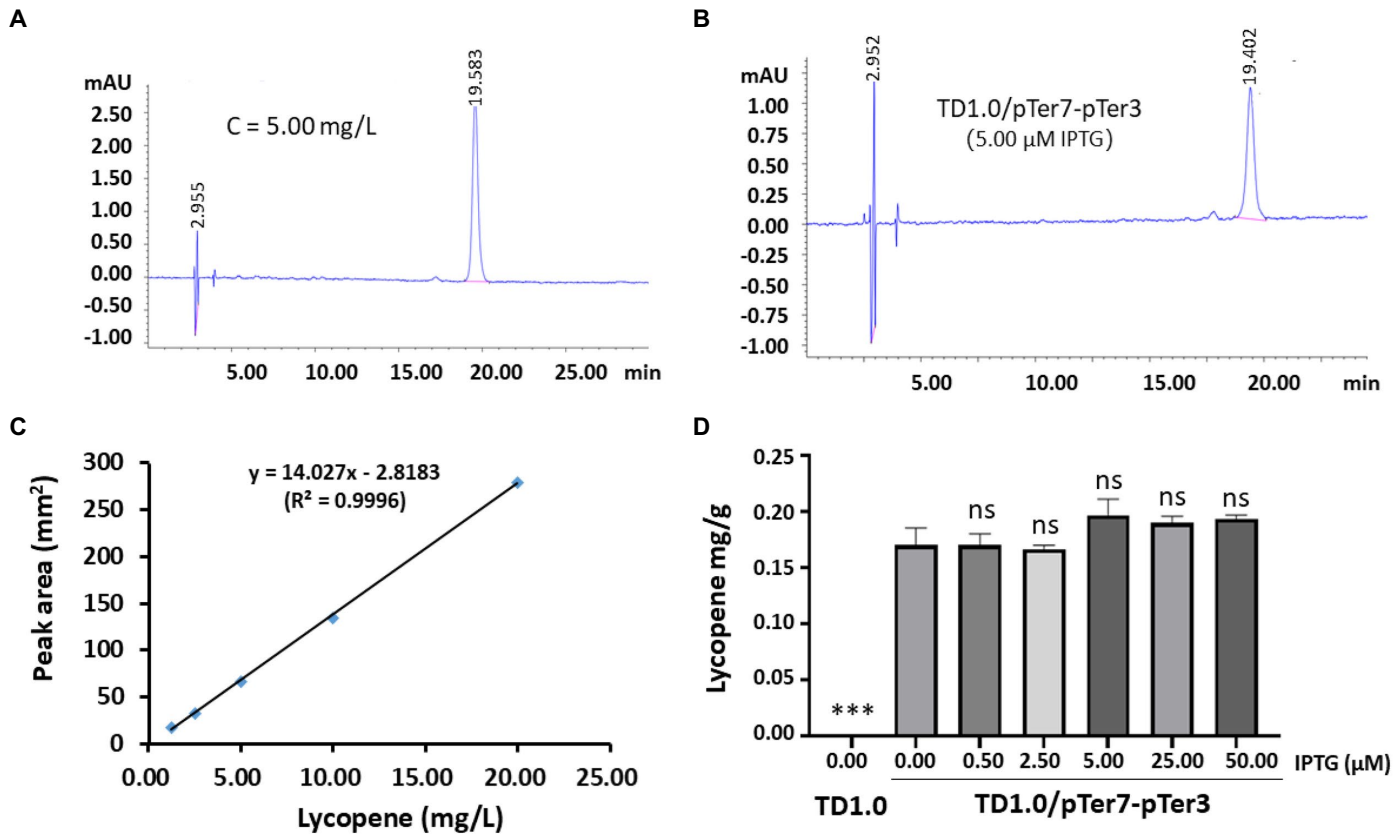
Production and characterization of lycopene in TD1.0/pTer7-pTer3 induced with different concentrations of IPTG. **(A)** The representative HPLC spectrum of the standard lycopene. A concentration of 5.00 mg/L lycopene was prepared, and the sample was detected by HPLC. The peak appeared at 20 min of elution. **(B)** The representative HPLC spectrum of the lycopene produced by TD1.0/pTer7-pTer3 after the induction of 5.00 μM IPTG. **(C)** Standard curve of lycopene. The lycopene standard was prepared and diluted with acetone for five concentration gradients (1.25, 2.50, 5.00, 10.00, and 20.00 mg/L). HPLC analysis was performed, and the peak areas were calculated for each concentration of lycopene. The curve was drawn using lycopene concentration against the relative peak areas. **(D)** Lycopene production in TD1.0/pTer7-pTer3 after induction with different concentrations of IPTG. *H. bluephagenesis* TD1.0/pTer7-pTer3 was cultured in a minimal medium supplemented with 30 g/L glucose as carbon source (60-MMG medium). For lycopene production, diverse concentrations of IPTG (0.00, 0.50, 2.50, 5.00, 25.00, and 50.00 μM) were added (*n* = 3 per concentration). All samples were obtained after 48 h cultivation at 37°C with shaking. Bacterial cells were harvested after culture, lysed, and subjected to HPLC analysis. The lycopene yield was calculated according to the standard curve and presented as milligrams per gram of DCW. The data were presented as mean ± standard derivation (SD). Statistical significances were calculated by one-way ANOVA and compared with TD1.0/pTer7-pTer3 without IPTG induction (0.00 IPTG). ns indicates no statistical significance; ^***^p < 0.001.

The genes of the MVA pathway in the pTer7 plasmid were under the control of two promoters, *P_Mmp1_* and *P_trc_*. They were regulated by the LacI repressor (Supplementary Figure 3A). In comparison, the expression of *SacrtE*, *SacrtI*, and *SacrtB* genes by pTer3 plasmid was constitutive (Figure 1B). *H. bluephagenesis* TD1.0/pTer7-pTer3 was cultured and induced by 5.00 μM IPTG to detect the potential lycopene production. The standard lycopene was purchased, and a specific HPLC peak was produced for 5.00 mg/L lycopene (Figure 2A). A similar HPLC peak was presented using the cell lysate sample from the engineered TD1.0/pTer7-pTer3 but not when the wild-type TD1.0 was used (Figure 2B). This finding suggested the production of lycopene in TD1.0/pTer7-pTer3.

Several concentrations of lycopene standard (1.25, 2.50, 5.00, 10.00, and 20.00 mg/L) were prepared, and a standard curve was drawn (Figure 2C). *H. bluephagenesis* TD1.0/pTer7-pTer3 was cultured. Lycopene production was induced with diverse concentrations of IPTG (0.00, 0.05, 2.50, 5.00, 25.00, and 50.00 μM) and analyzed *via* HPLC. The results showed that the wild-type TD1.0 produced no detectable lycopene. A maximum yield of approximately 0.20 mg/g (DCW) was achieved in TD1.0/pTer7-pTer3 under the induction of 5.00 μM IPTG (Figure 2D). Comparable lycopene production was observed in TD1.0/pTer7-pTer3 induced with different IPTG concentrations (Figure 2D). These data indicated that the leak expression of pTer7 can occur, or the intrinsic MEP pathway of *H. bluephagenesis* contributes to lycopene biosynthesis.

### Change in the downstream module genes increased the lycopene production in *Halomonas bluephagenesis*

**Figure 3:**
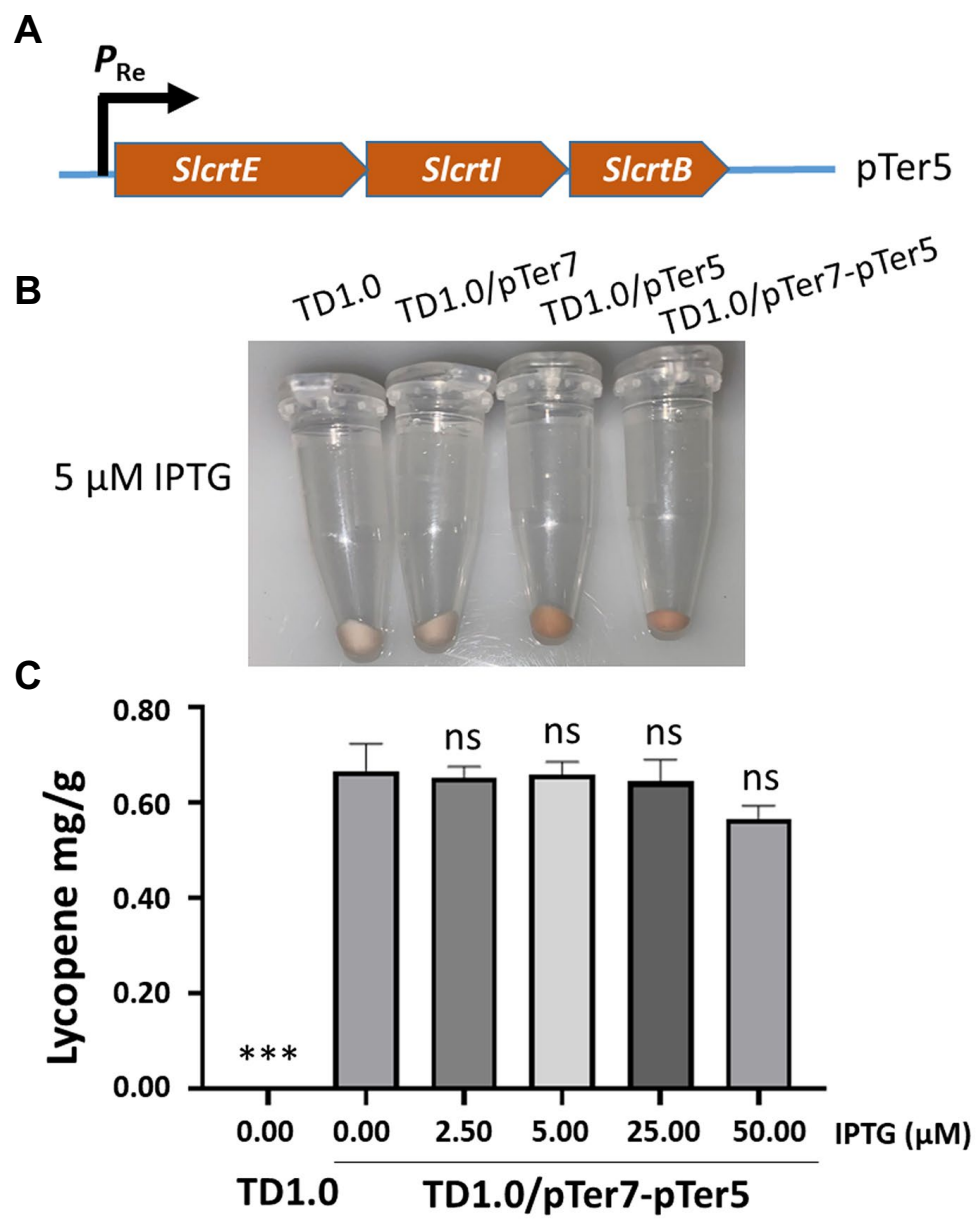
Change in downstream module genes to enhance the production of lycopene. **(A)** Schematic diagram of the construction of the pTer5 plasmid carrying MVA pathway downstream genes derived from *S. lividans*. **(B)** Cells were grown in a minimal medium supplemented with 30 g/L glucose as carbon source. Cell pellets of *H. bluephagenesis* strains TD1.0, TD1.0/pTer7, TD1.0/pTer5, and TD1.0/pTer7-pTer5 after induction with 5.00 μM of IPTG. **(C)** Lycopene production in TD1.0/pTer7-pTer5 after induction with the specified IPTG concentrations. The experiments were repeated three times for each concentration of IPTG induction. Cells were grown in a minimal medium supplemented with 30 g/L glucose as carbon source. All samples were obtained after 48 h cultivation at 37°C with shaking. The lycopene yield was calculated using the standard curve and presented as mg per gram of dried cell weight (DCW). The data were presented as mean ± SD. Statistical significance was calculated by One-way ANOVA and compared to TD1.0/pTer7-pTer5 without IPTG induction; ns indicates no statistical significance, ^***^p < 0.001.

The biosynthesis pathway genes derived from various organisms may result in different yields of chemicals in the engineered bacteria (Rad et al., 2012; Jiang et al., 2021). The downstream genes, including *crtE*, *crtI*, and *crtB*, for lycopene biosynthesis in pTer3 were replaced with those from *S. lividans*. The resultant plasmid pTer5 (Figure 3A) was transformed into *H. bluephagenesis* TD1.0 and *H. bluephagenesis* TD1.0 harboring pTer7, respectively. Notably, the transformation of pTer5 alone into *H. bluephagenesis* TD1.0 resulted in the production of red colors and 0.3 mg/g DCW lycopene (Figure 3B; Supplementary Figure 5). It was shown that the intrinsic MEP pathway of *H. bluephagenesis* contributes to the lycopene biosynthesis. The engineered TD1.0/pTer7-pTer5 also presented red colors after induction with 5.00 μM IPTG at 37°C for 48 h (Figure 3B). The lycopene yield in TD1.0/pTer7-pTer5 achieved 0.70 mg/g DCW (Figure 3C), about 3.5 times higher than that produced by TD1.0/pTer7-pTer3. These data suggested that the change in the downstream pathway genes contributes to the increase in lycopene production.

### Optimization of the promoters controlling the MVA pathway enhanced the lycopene production in *Halomonas bluephagenesis*

**Figure 4:**
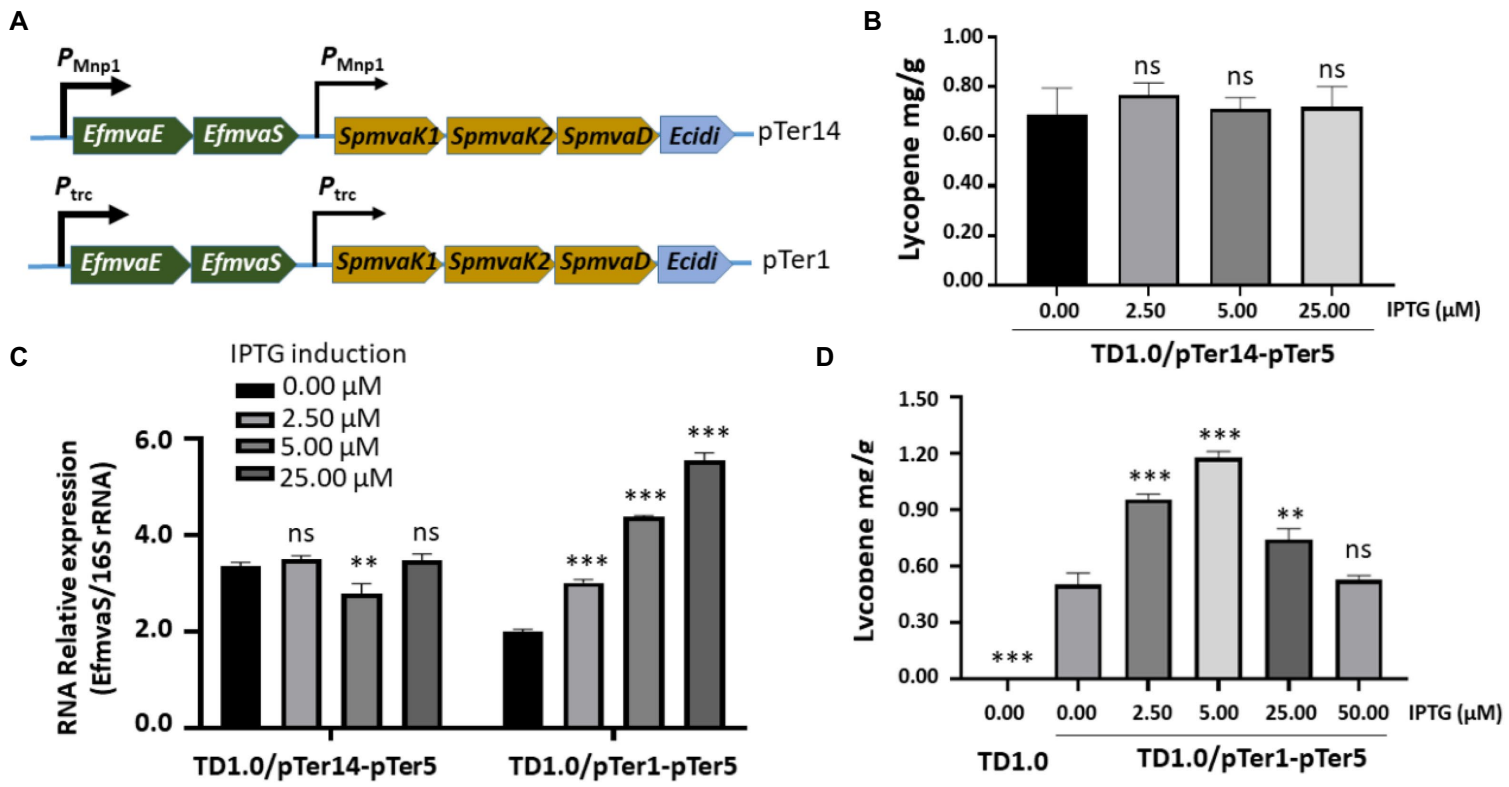
Optimizing promoters for enhanced lycopene synthesis. **(A)** Schematic diagram of the construction of the pTer14 and pTer1 plasmids carrying genes involved in the MVA pathway. The promoters that regulate the expression of MVA pathway genes were indicated. HPLC analysis of lycopene production from TD1.0/pTer14-pTer5 **(B)** and TD1.0/pTer1-pTer5 **(D)** after induction with different concentrations of IPTG. TD1.0 without IPTG induction served as negative control. The experiments were repeated three times for each concentration of IPTG induction. Cells were grown in a minimal medium supplemented with 30 g/L glucose as carbon source. All samples were obtained after 48 h cultivation at 37°C with shaking. The lycopene yield was analyzed by HPLC, calculated using the standard curve, and presented as mg per gram of dried cell weight (DCW). **(C)** RT-qPCR detection of *EfmvaS* expression in TD1.0/pTer14-pTer5 and TD1.0/pTer1-pTer5 cells with or without IPTG induction. The transcript level of the target gene was normalized to that of the 16S rRNA gene. The experiment was repeated at least three times. The data were presented as mean ± SD. Statistical significance was calculated by One-way ANOVA and compared to the engineered bacteria that did not receive IPTG induction; ns indicates no statistical significance, ^**^p < 0.01, and ^***^p < 0.001.

Lycopene production in both TD1.0/pTer7-pTer3 and TD1.0/pTer7-pTer5 seemed to tolerate IPTG induction (Figures 2D, 3C). The decreased lycopene yield was observed with the highest IPTG treatment. We hypothesized that the dual promoters (*P_Mmp1_*/*P_trc_*, strong/weak) constructed in the pTer7 plasmid may disturb the expression balance of genes involved in the MVA pathway of lycopene biosynthesis in *H. bluephagenesis.* Therefore, we set out to promoter optimization. On the basis of the pTer7 plasmid, the *P_trc_* promoter from *E. coli* was replaced with the phage MmpI promoter (*P_Mmp1_*) to generate pTer14 (Figure 4A top panel). After the transformation, *H. bluephagenesis* TD1.0/pTer14-pTer5 was subjected to fermentation analysis. The results demonstrated that the maximum production of lycopene in TD1.0/pTer14-pTer5 was 0.75 mg/g DCW (2.5 μM IPTG; Figure 4B), which was similar to that produced by TD1.0/pTer7-pTer5 (Figure 3C).

RT-qPCR revealed that replacing *P_trc_* with *P_Mmp1_* did not improve the reaction of TD1.0/pTer14-pTer5 to IPTG induction (Figure 4C). Therefore, we considered that a weak promoter combination may achieve good yields. Thus, the pTer1 plasmid that carried two *P_trc_* promoters was used (Figure 4A bottom panel). After the transformation into *H. bluephagenesis* TD1.0/pTer5, the expression of *EfmvaS* gene in the engineered TD1.0/pTer1-pTer5 responded well to IPTG induction (Figure 4C). The pellet colors of TD1.0/pTer1-pTer5 increased by elevating the IPTG treatment up to 5 μM (Supplementary Figure 6). HPLC analysis showed that the maximum lycopene production in TD1.0/pTer1-pTer5 achieved 1.22 mg/g DCW (5.00 μM IPTG; Figure 4D), approximately 1.7- and 6.1-fold higher than that from TD1.0/pTer7-pTer5 and TD1.0/pTer7-pTer3, respectively (Figures 3C, 4B). In summary, optimizing downstream gene sources and a weak promoter combination can synergistically improve lycopene production in the engineered *H. bluephagenesis.*

## Discussion

As an effective antioxidant, lycopene has been widely used in medicine and cosmetics (Rad et al., 2012). Metabolic engineering is a powerful approach for lycopene production with diverse microbial cell factories (Rad et al., 2012; Li et al., 2019). The MEP pathway harbored by bacteria and algae is naturally long, slightly efficient, and vaguely productive for lycopene production owing to the control mechanisms present in the native host (Martin et al., 2003). The high-efficiency MVA synthetic pathway usually carried by fungi or bacteria is attractive for manipulating metabolic engineering, resulting in varied lycopene production in diverse cell hosts (5–198 mg/g DCW, Supplementary Table 3). The introduction of the *crtE*, *crtB*, and *crtI* genes derived from *Erwinia herbicola*, *idi* gene from *Bacillus licheniformis*, and a heterologous MVA pathway (*mvaA*, *mvaS*, *mvaD*, *mvaK1*, and *mvaK2* from *S. pneumoniae* and *phbA* from *Ralstonia eutropha*) into *E. coli* resulted in 198 ± 3 mg/g DCW lycopene yield (Rad et al., 2012). However, the endotoxins and toxic enzymes with potential pathogenicity of bacterial chassis cells, such as *E. coli* and *Staphylococcus aureus*, limited their commercial production of lycopene (Yuan et al., 2018). The non-carotenogenic *H. bluephagenesis* is capable of growing at high concentrations of NaCl (60 g/L) and alkaline pH (8.5), thus, avoiding possible contamination by other microorganisms even under open unsterile conditions (Jiang et al., 2021). As a result, *H. bluephagenesis* based fermentations have reduced cost and complexity. In the present study, a heterogeneous lycopene biosynthetic pathway was generated, and metabolic optimization was performed to explore the nonpathogenic and environmentally friendly host *H. bluephagenesis* for microbial lycopene production and improvement.

An MVA pathway was heterogeneously constructed in *H. bluephagenesis* TD1.0. The results demonstrated that the engineered *H. bluephagenesis* could produce a certain amount of lycopene (Figure 2). In our preliminary design, the downstream genes of the lycopene biosynthetic pathway were obtained from *S. avivatus*, and the resultant strain TD1.0/pTer7-pTer3 only produced 0.2 mg/g of lycopene. The genes involved in lycopene biosynthesis among microorganisms are abundant. Their activities may vary in diverse sources and chassis hosts (Singh and Goyal, 2008; Reynaud et al., 2011). Replacing the downstream genes in plasmid pTer3 with those from *S. lividans* increased lycopene production by nearly three times (Figure 3C). On the basis of pTer7 with a promoter combination of *P_Mnp1_* and *P_trc_* (strong/weak), we found that the lycopene yield in TD1.0/pTer7-pTer3 or TD1.0/pTer7-pTer5 without IPTG induction was comparable with that of the bacteria treated with different IPTG concentrations (Figures 2D, 3C). Similar results were observed in the case of plasmid pTer14 that carried *P_Mmp1_* and *P_Mmp1_* promoter combination (strong/strong; Figure 4B). The reasons for this phenomenon were unclear. One possible explanation is that a certain leakage expression of the strong *P_Mmp1_* promoter occurs in the host *H. bluephagenesis* TD1.0. The RT-qPCR results revealed that the expression level of the *EfmvaS* gene under the control of *P_Mmp1_* in TD1.0/pTer14-pTer5 without IPTG treatment was similar to that in IPTG-induced bacteria (Figure 4C). Increasing the promoter strength may also lead to the enhancement of bacterial metabolic pressure or disturbance of host metabolic balance (Ruther et al., 1997). Considering the effect of the strength promoter, we tested whether a weak promoter combination can reverse the IPTG-induced reaction. As expected, the two *P_trc_* promoters (weak/weak combination) carrying pTer1 plasmid in TD1.0/pTer1-pTer5 responded well to the IPTG induction (Figure 4C). The lycopene production increased along with the elevation of IPTG concentrations to 5.00 μM. Thereafter, it decreased for both 25.00 and 50.00 μM of inducer (Figure 4D). These data suggested that the increase in promoter strength may not consistently achieve a high level of chemical production. Moreover, a suitable promoter combination is important and should be investigated further.

In conclusion, the heterogeneous MVA pathway was constructed in *H. bluephagenesis* to enable lycopene production. The change in downstream gene sources involved in the lycopene biosynthetic pathway and optimization of weak/weak promoter combination could synergistically promote lycopene production to 1.22 mg/g DCW in our experimental condition. In our study, a novel primary *Halomonas* host is provided for further metabolic engineering operations, such as MVA pathway optimization and by-pass pathway block, which will be further investigated in the near future.

## Data availability statement

The original contributions presented in the study are included in the article/Supplementary material, further inquiries can be directed to the corresponding authors.

## Author contributions

X-RJ and XR designed the study, interpreted the data, and revised the manuscript. QS acquired the data and prepared the manuscript. QS, PC, JS, and YuZ conceived the study and acquired the data. YaZ analyzed and interpreted the data. X-RJ supervised the project and obtained funding. All authors contributed to the article and approved the submitted version.

## Funding

This work was supported by the National Natural Science Foundation of China (grant nos. 32171415 and 31900045).

## Conflict of interest

The authors declare that the research was conducted in the absence of any commercial or financial relationships that could be construed as a potential conflict of interest.

## Publisher’s note

All claims expressed in this article are solely those of the authors and do not necessarily represent those of their affiliated organizations, or those of the publisher, the editors and the reviewers. Any product that may be evaluated in this article, or claim that may be made by its manufacturer, is not guaranteed or endorsed by the publisher.
