# Supplementary material for: Engineering a mevalonate pathway in Halomonas bluephagenesis for the production of lycopene
Source: Front Microbiol. 2023 Jan 16;13:1100745. doi: 10.3389/fmicb.2022.1100745 (PMC9885113; doi:10.3389/fmicb.2022.1100745)
Supplement: Supplementary file 1 [file Data_Sheet_1.pdf]

SUPPLEMENTARY MATERIALS:

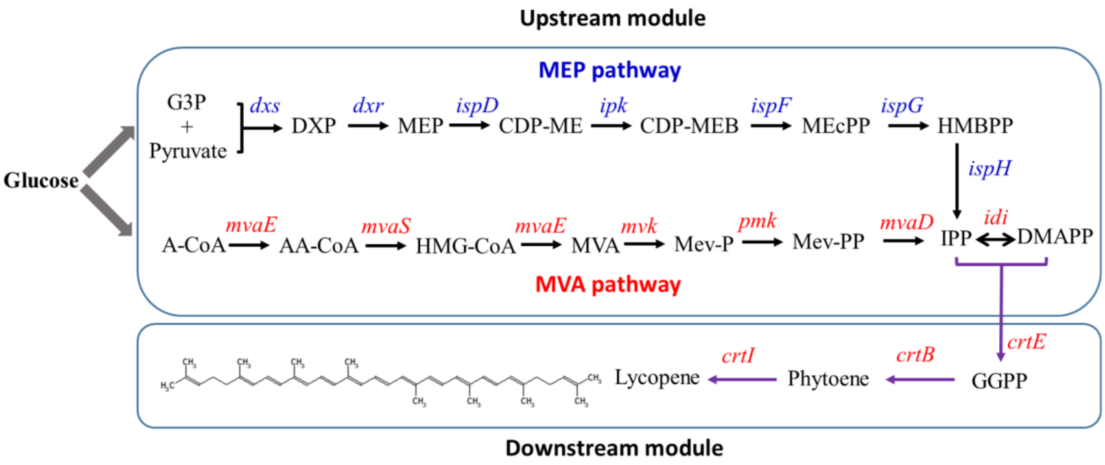

Supplementary Figure 1 | Schematic diagram of lycopene production in *Halomonas bluephagenesis*. The genes involved in the internal MEP pathway are indicated by blue texts while the heterogeneous genes overexpressed are indicated in red texts. The heterogeneous genes *mvaE* and *mvaS* from *E. faecalis*, *mvk*, *pmk*, and *mvaD* from *S. pneumoniae*, *idi* from *E. coli* were selected to construct a heterologous MVA pathway in *H. bluephagenesis* TD1.0. Pathway intermediates: G3P, glyceraldehyde 3-phosphate; DXP, 1-deoxy-D-xylulose 5-phosphate; MEP, 2-C-methyl-D-erythritol 4-phosphate; CDP-ME, 4-diphosphocytidyl-2-C-methyl-D-erythritol; CDP-MEP, 4-diphosphocytidyl-2-C-methyl-D-erythritol 2-phosphate; MEcPP, 2-C-methyl-D-erythritol 2,4-cyclopyrophosphate; HMBPP, 1-hydroxy-2-methyl-2-(E)-butenyl 4-pyrophosphate; IPP, isopentenyl pyrophosphate; DMAPP, dimethylallyl pyrophosphate; A-CoA, acetyl-CoA; AA-CoA, acetoacetyl-CoA; HMG-CoA, hydroxymethylglutaryl-CoA; MVA, mevalonate; Mev-P, mevalonate 5-phosphate; Mev-PP, mevalonate pyrophosphate; GGPP, geranylgeranyl diphosphate. Enzyme abbreviations: MvaE, acetyl-CoA acetyltransferase/hydroxymethylglutaryl-CoA reductase; MvaS, hydroxymethylglutaryl-CoA synthase; Mvk, mevalonate kinase; Pmk, MVAP kinase; MvaD, MVAPP decarboxylase; DXS, DXP synthase; DXR, DXP reductoisomerase; IspD, CDP-ME cytidylyltransferase; IspE, CDP-ME kinase; IspF, MEC synthase; IspG, HMBPP synthase; IspH, HMBPP reductase; IDI, isopentenyl diphosphate isomerase; CrtE, GGPP synthase; CrtB, phytoene synthase; and CrtI, phytoene desaturase.

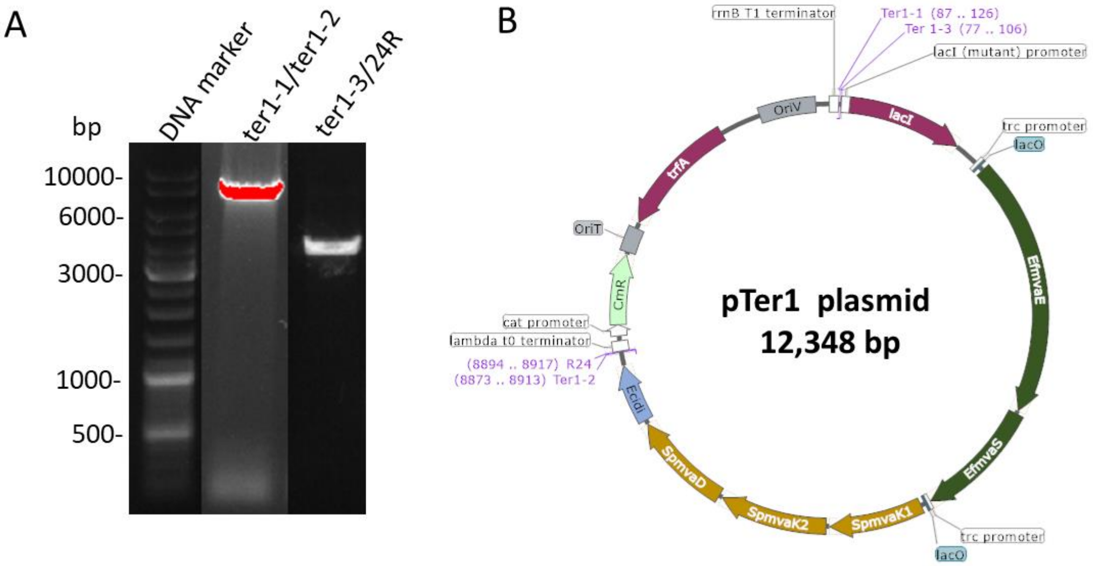

Supplementary Figure 2 | Construction of plasmid pTer1 carrying a heterogeneous MVA pathway for lycopene production. **(A)** PCR fragments obtained from plasmids pSEVA321 and pMVA3 (Jervis et al., 2019) with the indicated primer pairs, respectively. The sizes of DNA marker were indicated on the left. **(B)** The map of pTer1 plasmid. The plasmid was constructed by Gibson Assembly (Gibson et al., 2009). A heterogeneous MVA pathway genes, including *Enterococcus faecalis* (*Ef*)-derived *mvaE* and *mvaS* genes, *Streptococcus pneumoniae* (*Sp*)-originated *mvaK1*, *mvaK2*, and *mvaD* genes, and *E. coli* (*Ec*)-derived *idi* gene, were indicated.

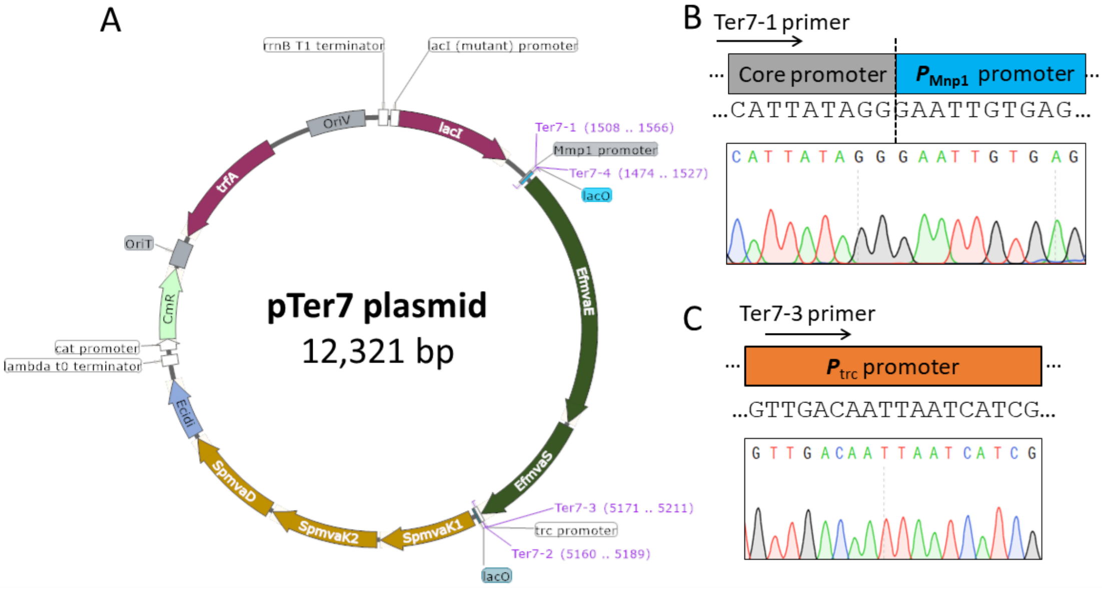

Supplementary Figure 3 | Construction of plasmid pTer7 carrying a heterogeneous MVA pathway for lycopene production. **(A)** The map of pTer7 plasmid. A heterogeneous MVA pathway genes, including *EfmvaE*, *EfmvaS*, *SpmvaK1*, *SpmvaK2*,

SpmvaD, and *Ecidi* genes, were indicated. **(B)** DNA sequencing revealed successful replacement of P_{Mnp1} promoter. **(C)** DNA representing the retained P_{trc} promoter in the pTer7 plasmid.

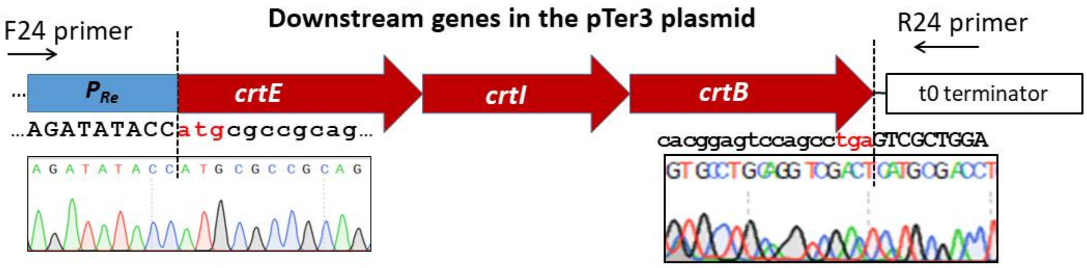

Supplementary Figure 4 | DNA sequencing showing the correct construction of downstream *crtE*, *crtI*, and *crtB* genes derived from *Streptomyces avermitilis* (*Sa*) in plasmid pTer3. The primers for sequencing were indicated. The start codon 'ATG' of the *crtE* gene and the stop codon 'TGA' of *crtB* gene were indicated as red texts.

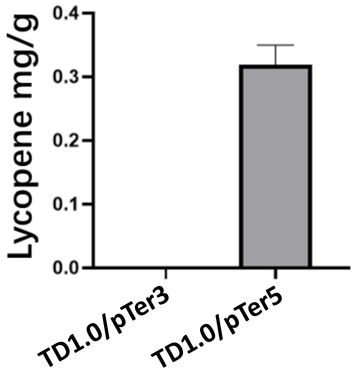

Supplementary Figure 5 | Comparison of downstream module metabolites between *H. bluephagenesis* TD1.0/pTer3 and *H. bluephagenesis* TD1.0/pTer5. Cells were grown in a minimal medium supplemented with 30 g/L glucose as carbon source. All samples were obtained after 48 h cultivation at 37 ℃ with shaking. The yield of lycopene was calculated according to the standard curve and presented as mg per gram of dried cell weight (DCW). The data were presented as mean ± SD.

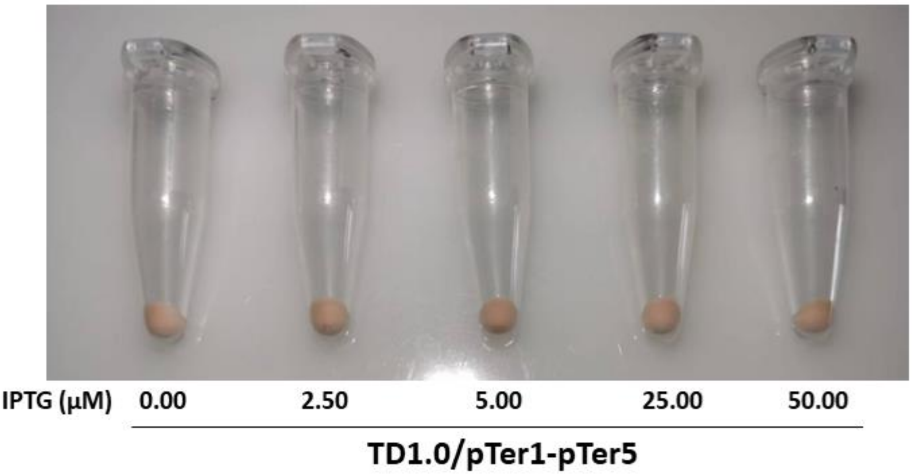

Supplementary Figure 6 | Cell pellets of engineered *H. bluephagenesis* strain TD1.0/pTer1-pTer5 induced with various concentrations of IPTG.

Supplementary Table 1 | Strains and plasmids used in this study

Strains and Plasmids	Description	Sources
**Strains**		
Halomonas bluphagenesis TD1.0	A novel T7-like RNA polymerase-integrated derivative of the wild-type *H. bluephagenesis* strain TD01	Zhao et al., 2017
E. coli S17-1	The donor *E. coli* used to perform the conjugative transformation. The genome contains the *tra* gene; *proA, thi-1*	Simon, 1984
TD1.0/pTer7-pTer3	TD1.0 carrying pTer7 and pTer3 plasmids	This work
TD1.0/pTer7-pTer5	TD1.0 carrying pTer7 and pTer5 plasmids	This work
TD1.0/pTer14-pTer5	TD1.0 carrying pTer14 and pTer5 plasmids	This work
TD1.0/pTer1-pTer5	TD1.0 carrying pTer1 and pTer5 plasmids	This work
**Plasmids**		
pSEVA321	Standard European Vector Architecture	Martínez-García et al., 2019
pMVA2	Carrying a heterogeneous MVA pathway genes, including *EfmvaE, EfmvaS, SpmvaK1, SpmvaK2, SpmvaD*, and *Ecidi* under the control of two *E. coli* P_{trc} promoters	Jervis et al., 2019
pSEV434	Standard European Vector Architecture	Martínez-García et al., 2019
pTer1	Two weak promoters (P_{trc}) controlled the expression of upstream genes in the conversation of acetyl-CoA to IPP/DMAPP. OriV/OriT, Cm^R, LacI	This work
pTer3	The constitutive promoter (P_{Re}) controlled the expression of downstream genes (*crtE*, *crtB*, and *crtI*) genes derived from *Streptomyces avivatus*. Kan^R, Spe^R	This work
pTer5	The constitutive promoter (P_{Re}) controlled the expression of downstream genes (*crtE*, *crtB*, and *crtI*) genes derived from *Streptomyces lividans*. Kan^R, Spe^R	This work

pTer7	A weak promoter (*P_{trc}*) and one strong promoter (*P_{Mnp1}*) controlled the expression of upstream genes in the conversation of acetyl-CoA to IPP/DMAPP. OriV/OriT, Cm^R, LacI	This work
pTer14	Two strong promoters (*P_{Mnp1}*) controlled the expression of upstream genes in the conversation of acetyl-CoA to IPP/DMAPP. OriV/OriT, Cm^R, LacI	This work

Supplementary Table 2 | Primers used in this study

Primers	Sequences (5'– 3')	Descriptions	Product sizes
Ter1-1	gatgcctttaattaaagcggacgtcgacaccatcgaatgg	Amplification of *lacI*, *EfmvaE*, *EfmvaS*, *SpmvaK1*, *SpmvaK2*, *SpmvaD*, *Ecidi* gene for pTer1 construction	8827 bp
Ter1-2	agggttttcccagtcacgacatgcctggagatccttactcg		
Ter1-3	ccgctttaattaaaggcatcaaataaaacg	Amplification of *OriV*, *OriT*, Cm^R for pTer1 construction	3561 bp
24R	gtcgtgactgggaaaaccctg		
Ter2-3	GGTATATCTCCTTATTAAAGTTAAACAAAATTATTTCTACAGG	Amplification of P_{Re} promoter for pTer3 construction	643bp
275-1	ggcgcgcccagctgtctag		
275-3	ctagacagctgggcgcgcc	Amplification of plasmid replicon for pTer3 construction	1789bp
Jxr53-7	cgctgcataaccctgcttcg		
Jxr53-5	gtcgacctgcaggcatgcaag	Amplification of resistance gene for pTer3 construction	550 bp
Jxr53-6	cgaagcagggttatgcagcg		
Ter3-1	CTTTAATAAGGAGATATACCatgcgccgcagcaaggcgac	Amplification of *SacrtEIB* for pTer3 construction	3845bp
Ter3-2	ttgcatgcctgcaggtcgactcatgcgacctcctcatgtg		
Ter5-1	CTTTAATAAGGAGATATACCgtgcggacaaccgagagtgag	Amplification of *SlcrtEIB* for pTer3 construction	3769bp
Ter5-2	ttgcatgcctgcaggtcgactcaccgcaccggtccctgcc		
Ter7-1	gcattatagggaattgtgagcgctcacaattattcaaaagatcttttaaggacgaaacg	Amplification of P_{Mmp1} promoter, *EfmvaE* and *EfmvaS* genes for pTer7 construction	3682bp
Ter7-2	cgatgattaattgtcaacagctttaattac		
Ter7-3	tgttgacaattaatcatcggctcgtataatgtgtggaattg	Amplification of *SpmvaK1*, *SpmvaK2*, *SpmvaD*, and *Ecidi* genes and plasmid replicon for pTer7 construction	8678bp
Ter7-4	ctcacaattccctataatgccacaaatatcatttcagaatatttgccagaaccg		
Ter14-1	CGCTCACAATTATTCAAAAGATCTTTTAAGGAC	Amplification of P_{Mmp1} promoter, *EfmvaE and EfmvaS* gene for pTer14 construction	3662bp
Ter14-2	CTCCCTATAATGCCACAAATATTTAATTACGATAGCTACGCACGG		
Ter14-3	ATTTGTGGCATTATAGGGAGAATTGTGAGCGCTCACAATTCAGGCTCCCATTTAACACG	Amplification of *SpmvaK1*, *SpmvaK2*, *SpmvaD*, *Ecidi* gene and resistance gene for pTer14 construction	4652bp
Jxr53-6	cgaagcagggttatgcagcg		
Ter14-4	CTTTTGAATAATTGTGAGCGCTC	Amplification of plasmid	4046bp

	ACAATTCTCCCTATAATGCCACA<br>AATATCATTTCAG	replicon and *lacI* gene for<br>pTer14 construction
Jxr53-7	cgctgcataaccctgcttcg	

Supplementary Table3 | Summary of lycopene production in engineered microorganisms.

Organism	Strategies	Culture condition	Lycopene production	Reference
H. bluephagenesis	Plasmid based overexpression of lycopene synthesis genes	Shake flask, 48h	1.22 mg/g DCW	This work
E. coli	Introduction of a heterologous mevalonate pathway, substrate optimization	Shake flask, 24h	198 ±3 mg/g DCW	Rad et al., 2012
E. coli	Pathway balancing	Fed-batch	34.3 mg/g DCW	Zhu et al., 2015
E. coli	Plasmid based overexpression of carotenoids synthesis genes	Shake flask	67 mg/g DCW	Xu et al., 2018
S. cerevisiae	Directed evolution and metabolic engineering	Fed-batch, 120h	24.41 mg/g DCW	Xie et al., 2015
S. cerevisiae	Pathway engineering	Fed-batch	55.56 mg/g DCW	Chen et al., 2016
Y. lipolytica	Two copies of HMG1, two copies of CrtI, and single copies of MVD1, EGR8, CrtB, and CrtE	Fed-batch, 10 days	21.1 mg/g DCW,	Schwartz et al., 2017
P. pastoris	Overexpression of HMGR and HMGS; overexpression of GGPPS; increasing the copy number of crt genes	fed batch, 136 h	9.319 mg/g of DCW	Zhang et al., 2020
M. circinelloides	ΔcrgA, negative regulator of carotenogenesis; utilization of a complex enriched medium	Shake flask, 5 days	5 mg/g of DCW	Nicolas-Molina et al., 2008
S. avermitilis	Seven synthetic promoters and insulator RiboJ were inserted upstream of crt gene cluster	Shake flask, 5 days	82 mg/g of DCW	Bai et al., 2015

References

Bai, C., Zhang, Y., Zhao, X., Hu, Y., Xiang, S., Miao, J., et al. (2015). Exploiting a precise design of universal synthetic modular regulatory elements to unlock the microbial natural products in *Streptomyces*. *Proc. Natl. Acad. Sci. U.S.A.* 112, 12181−12186. doi: 10.1073/pnas.1511027112

Chen, Y., Xiao, W. H., Wang, Y., Liu, H., Li, X., and Yuan, Y. J. (2016). Lycopene

overproduction in *Saccharomyces cerevisiae* through combining pathway engineering with host engineering. *Microb. Cell Fact.* 15:113. doi: 10.1186/s12934-016-0509-4

Gibson, D. G., Young, L., Chuang, R. Y., Venter, J. C., Hutchison, C. A. 3rd, and Smith, H. O. (2009). Enzymatic assembly of DNA molecules up to several hundred kilobases. *Nat. Methods.* 6, 343–345. doi: 10.1038/nmeth.1318

Jervis, A. J., Carbonell, P., Vinaixa, M., Dunstan, M. S., Hollywood, K. A., Robinson, C. J., et al. (2019). Machine learning of designed translational control allows predictive pathway optimization in *Escherichia coli*. *ACS Synth. Biol.* 8, 127–136. doi: 10.1021/acssynbio.8b00398

Martínez-García, E., Goñi-Moreno, A., Bartley, B., McLaughlin, J., Sánchez-Sampedro, L., Pascual del Pozo, H., et al. (2020). SEVA 3.0: an update of the Standard European Vector Architecture for enabling portability of genetic constructs among diverse bacterial hosts. *Nucleic Acids Res.* 48, D1164-D1170. doi: 10.1093/nar/gkz1024

Nicolas-Molina, F. E., Navarro, E., Ruiz-Vazquez, R. M. (2008). Lycopene over-accumulation by disruption of the negative regulator gene *crgA* in *Mucor circinelloides*. *Appl. Microbiol. Biotechnol.* 78, 131−137. doi: 10.1007/s00253-007-1281-5.

Rad, S. A., Zahiri, H. S., Noghabi, K. A., Rajaei, S., Heidari, R., and Mojallali, L. (2012). Type 2 IDI performs better than type 1 for improving lycopene production in metabolically engineered *E. coli* strains. *World J. Microbiol. Biotechnol.* 28, 313–321. doi: 10.1007/s11274-011-0821-4

Simon, R. (1984). High frequency mobilization of gram-negative bacterial replicons by the in vitro constructed Tn5-Mob transposon. *Mol. Gen. Genet.* 196, 413–420. doi: 10.1007/BF00436188.

Schwartz, C., Frogue, K., Misa, J., and Wheeldon, I. (2017). Host and pathway engineering for enhanced lycopene biosynthesis in *Yarrowia lipolytica*. *Front. Microbiol.* 8, 11. doi: 10.3389/fmicb.2017.02233

Xie, W., Lv, X., Ye, L., Zhou, P., and Yu, H. (2015). Construction of lycopeneoverproducing *Saccharomyces cerevisiae* by combining directed evolution and metabolic engineering. Metab. Eng. 30, 69–78. doi: 10.1016/j.ymben.2015.04.009

Xu, J., Xu, X., Xu, Q., Zhang, Z., Jiang, L., and Huang, H. (2018). Efficient production of lycopene by engineered *E. coli* strains harboring different types of plasmids. *Bioprocess. Biosyst. Eng.* 41, 489–499. doi: 10.1007/s00449-017-1883-y

Zhao, H, Zhang, H. M., Chen, X., Li, T., Wu, Q., et al. (2017). Novel T7-like expression systems used for *Halomonas*. *Metab. Eng.* 39, 128–140. doi: 10.1016/j.ymben.2016.11.007

Zhu, F., Lu, L., Fu, S., Zhong, X., Hu, M., et al. (2015). Targeted engineering and scale up of lycopene overproduction in *Escherichia coli*. *Process Biochem.* 50, 341–346. doi: 10.1016/j.procbio.2014.12.008

Zhang, X., Wang, D., Duan, Y., Zheng, X., Lin, Y., and Liang, S. (2020). Production

of lycopene by metabolically engineered *Pichia pastoris*. *Biosci. Biotechnol. Biochem.* 84, 463−470. doi: 10.1080/09168451.2019.1693250.
